# Dynamic regulation of autophagy during Semliki Forest virus infection of neuroblastoma cells

**DOI:** 10.1099/jgv.0.002086

**Published:** 2025-03-05

**Authors:** Robert J. Stott-Marshall, Craig McBeth, Thomas Wileman

**Affiliations:** 1 One Virology, School of Veterinary Science and Medicine, University of Nottingham, Nottingham, LE12 5RD, UK; 2 School of Health, Leeds Beckett University, Leeds, LS1 3HE, UK; 3 School of Health and Life Sciences, Teesside University, Middlesbrough, TS1 3BX, UK; 4 Norwich Medical School, University of East Anglia, Norwich, NR4 7TJ, UK

**Keywords:** alphavirus, autophagy regulation, mTOR, neurons, ssRNA virus, *Togaviridae*

## Abstract

Autophagy can defend against infection by delivering viruses to lysosomes for degradation. Semliki Forest virus (SFV) is a positive-sense, single-stranded RNA virus of the alphavirus genus which has been used extensively as a model for arbovirus infection and neuronal encephalitis. Here, we show that autophagy is suppressed during the early hours of SFV infection of neurons. We also show that a switch between autophagy suppression and upregulation between the early and later stages was mediated through modulation of the mammalian target of rapamycin (mTOR) activity during infection. At later stages of infection, autophagosomes colocalize with SFV nonstructural proteins suggesting the formation of a platform for virus replication. Inhibition of mTOR by torin reduced infectious virus production and intracellular virus gene expression while improving cell survival during infection. The results suggest that autophagy is suppressed early during infection of neurons to increase cell survival and then upregulated at later times to facilitate replication. This biphasic regulation of autophagy seen for SFV may be important for other arboviruses, and knowledge about the regulation of autophagy by alphaviruses may be useful for the development of antiviral therapies.

## Introduction

Autophagy is an evolutionarily conserved process that promotes cell survival by delivering long-lived proteins or damaged organelles to lysosomes for degradation. Substrates for autophagy are sequestered into double-membrane vesicles termed autophagosomes that are delivered to lysosomes and then degraded by lysosomal proteases [1]. Autophagy also plays a key role in the degradation of intracellular pathogens, and many pathogens may have evolved the ability to inhibit autophagy to enhance their production [2].

Semliki Forest virus (SFV) is a single-stranded, positive-sense RNA virus of the *Alphavirus* genus in the family *Togaviridae*. The RNA genome has two ORFs that code for two polyproteins. The nonstructural polyprotein is situated in the 5′ two-thirds of the genome, while the structural polyprotein is carried on a subgenomic 26S promoter and is transcribed from the 3′ end. Entry into the host cell is via clathrin-mediated endocytosis. Once inside the cell, the virus is uncoated, and the genome is released. Replication occurs in membranous compartments termed replication complexes [3,4]. Assembly of these replication complexes is directed by nonstructural protein 1 (nsP1) and occurs at the cytosolic surface of late endosomes and lysosomes [5]. The virus recruits membranes of the endoplasmic reticulum to these sites of replication [6]. SFV replication complexes are transported along a multi-step vesicular transport pathway that relies heavily on microtubules and results in the formation of large acidic vacuoles that are concentrated around the microtubule-organizing centre (MTOC) [7].

There is contrasting evidence for the role of autophagy during infection with alphaviruses. In the Chikungunya virus (CHIKV), it has been shown that key components of autophagy may direct viral proteins to autophagosomes for degradation, but this effect is negated through the use of autophagosomal membranes as sites for replication complexes which leads to enhanced virus production [8,10]. Conversely, there is evidence that autophagy is activated by macrophage scavenger receptor 1 during CHIKV infection of mice, which restricts the virus infection [11], and that genetic knockout of autophagy genes enhances CHIKV replication as well as apoptosis [8,12]. It has also been reported that autophagy has a protective effect in cells infected with Sindbis virus (SINV) through targeted clearance of viral proteins, but that independent knockout of autophagy-related genes has no effect on the production of infectious virus from these cells [13,14]. Further investigation of this protective effect *in vitro* and *in vivo* has shown that increased autophagy and, therefore, an increase in the key autophagy protein Beclin-1 result in an increase in Beclin-1 interaction with the anti-apoptotic protein Bcl-2, thereby enhancing cell survival [15,16]. Autophagosomes have been shown to accumulate during the late stages of infection with SFV and are dependent on the expression of the viral glycoproteins [17]. However, SFV and CHIKV have also been shown to activate PI3K-Akt-mTOR activity early in infection, which may lead to an inhibition of autophagy [18]. This contrasting evidence suggests that autophagy may play a dynamic role during alphavirus infection and that it may be induced or inhibited at different stages of the virus life cycle.

Although the pathogenesis of alphaviruses occurs once the infection passes the blood–brain barrier and into the nervous system [19,20], very little is known about the relationship between autophagy and virus infection in neurons. The importance of strict regulation of the turnover of cytosolic contents in these cells suggests that autophagy may be regulated differently in neuronal cells than in non-neuronal cells [21,24]. It has also been reported that the mechanism of autophagy regulation in neurons may differ at least in part from that of non-neuronal cells [25]. The neuronal response to SFV infection has been shown to differ during the early and late stages of infection [26]. However, there are currently no published studies on autophagy during SFV infection of neurons. This prompted us to study autophagy following infection of cultured human neurons with SFV. The results show that SFV inhibits autophagy early during infection but increases autophagosome formation at later stages. We also show that chemical activation of autophagy early in infection substantially lowers virus yield. Taken together, the results show that SFV dynamically regulates autophagy during the early and late stages of infection. It also suggests that virus-mediated inhibition of autophagy early during infection allows SFV to establish infection in neuronal cells and that chemical induction of autophagy may have an antiviral role.

## Methods

### Cell lines

The human neuroblastoma cell line SK-N-SH (ECACC, Public Health England) was maintained in minimum essential medium (MEM) (Biowest) supplemented with 10% heat-inactivated foetal calf serum (HIFC) (Sigma-Aldrich, UK). Human neuroblastoma cell line IMR-32 (ECACC, Public Health England) was maintained in MEM supplemented with 10% HIFC and 0.1 mM MEM Eagle non-essential amino acids (NEAA) (LONZA). Wild-type and Atg5 -/- knockout cells were grown in Dulbecco’s Modified Essential Medium (Biowest) supplemented with 10% HIFC. These cell-specific media formulations are herein referred to as complete media. Cell lines were grown in a humidified 37 °C incubator with 5% CO2.

### Virus infection and UV inactivation

Wild-type SFV4 was derived from the infectious clone pSP6-SFV4 as described by Liljeström *et al*. [27]. Virus stocks were produced by infecting human cervical carcinoma cells (HeLa; ECACC, Public Health England) and incubating for 24 h in MEM supplemented with 2% HIFC (Biowest), 0.1 mM MEM Eagle NEAA (LONZA), 100 U ml^−1^ penicillin and 100 μg ml^−1^ streptomycin (Biowest) or until complete cytopathic effect (CPE) was observed. The supernatant was stored at −80 °C and used for virus infections. For experimental infection, SFV was diluted in serum-free media specific to each cell line as indicated above to the multiplicity of infection (MOI) as indicated in each experiment. Cells were incubated with virus inoculum for 1 h at 37 °C before the inoculum was removed and cells were overlaid with complete media.

Viral titres were determined by plaque assay in HeLa cells as previously described [28,29]. Briefly, HeLa cells were seeded into 6-well plates and infected with serial dilutions of virus samples for 1 h at 37 °C. Cells were then overlaid with EMEM containing 5% HIFC and 0.9% UltraPure™ low melting point agarose (Thermo Fisher, UK) and incubated for 48 h at 37 °C. Cells were fixed with 4% formaldehyde, and plaques were visualized by staining monolayers with 1% crystal violet.

Infectious virus stocks were inactivated by exposure to UV light using a CL-1000 Crosslinker (UVP). Virus stocks were exposed to UV light intensity of 0.72 J cm^−2^. Virus inactivation was confirmed by adding a UV-inactivated virus to cell monolayers and examining for CPE.

### Autophagy drug treatments

A 1 mM stock solution of Torin2 (Selleckchem, UK) was prepared in DMSO and used at a final concentration of 2 µM in complete media. A 100 mM stock solution of chloroquine (CHQ) (Sigma-Aldrich, UK) was prepared in water, filter sterilized and used at a final concentration of 100 µM in complete media.

### MTT colorimetric assay

The 3-[4,5-dimethylthiazol-2-yl]-2,5-diphenyltetrazolium bromide (MTT) (thiazolyl blue) colorimetric assay was used to measure cell proliferation. Cells were seeded into 96-well plates and treated as specified by each experiment. Cells were incubated with a final concentration of 0.5 mg ml^−1^ MTT (Sigma-Aldrich, UK) diluted in MEM for 4 h at 37 °C. Culture media were removed, and formazan crystals were solubilized with DMSO. Absorbance was measured at 540/690 nm, and percentage cell proliferation was expressed using the following formula:

$100\;x\;\left(\frac{absorbance\;of\;experimental\;cells}{absorbance\;of\;control\;cells}\right)$

### Western blot

Whole-cell lysates were denatured in Laemmli buffer at 100 °C for 5 min before separation by SDS-PAGE using 4% stacking and 15% separating gels. Protein was then transferred to 0.2 µm Amersham™ Hybond™ PVDF membrane (VWR, UK) using a Trans-Blot® Turbo™ Transfer System (Bio-Rad, UK). Membranes were blocked for 1 h at room temperature in a blocking buffer (5% w/v BSA (Sigma-Aldrich, UK) in TBS with 0.5% Tween®20 (TBS-T). Primary antibodies used were rabbit anti-LC3B (diluted 1:3,000), rabbit anti-p62/SQSTM (1:1,000), rabbit anti-p70S6 kinase (1:1,000), rabbit anti-phospho-p70S6 kinase (Thr421/Ser424; Cell Signaling Technologies, UK; 1:1,000), mouse anti-actin (Abcam, UK; 1:400) and rabbit anti-SFV nsP2 (a gift from Andres Merits, University of Tartu; 1:10,000). These were diluted in blocking buffer and incubated at 4 °C overnight. AffiniPure anti-Rabbit (diluted 1:20,000) and anti-Mouse (1:40,000) secondary antibodies conjugated with HRP (Jackson ImmunoResearch, UK) were diluted in blocking buffer and incubated at room temperature for 1 h. Bands were developed with ECL reagent and visualized by chemiluminescence detection using a GeneGnome XRQ chemiluminescence detection system (Syngene, UK). Relative protein levels were quantified using densitometric analysis of bands in ImageJ software.

### Immunofluorescence microscopy

Cells were grown on methanol-sterilized glass coverslips and then infected with SFV. Cells were fixed with 4% paraformaldehyde at room temperature and permeabilized with 100% ice-cold methanol at −20 °C for 10 min. Coverslips were blocked with a blocking buffer of 2% BSA in PBS for 1 h at room temperature. Primary antibodies used were anti-LC3B conjugated with DyLight 550 (Thermo Fisher, UK; diluted 1:2,000), rabbit anti-SFV nsP1 (1:10,000), rabbit anti-SFV nsP3 (1:10,000) and anti-SFV capsid (1:10,000; Andres Merits, University of Tartu) and were diluted with blocking buffer and incubated at 4 °C overnight. The secondary antibody, anti-rabbit AlexaFluor488, was diluted with blocking buffer and incubated at room temperature for 40 min. Coverslips were mounted using Fluoroshield™ with a DAPI mounting medium (Sigma-Aldrich, UK). Imaging of multiple *z*-axis positions was performed using a Zeiss AxioImager M2 system, and images were deconvoluted using the Zeiss Zen software deconvolution module.

For quantification of LC3-positive punctae, images of only the DyLight 550 channel were selected at random and spots were counted in ImageJ. The data were tabulated in Excel before revealing other channels and identifying infection status. Mean fluorescence intensity (MFI) was also calculated using ImageJ by selecting drawing regions of interest around SFV nsP1 positive cells and comparing them with SFV nsP1 negative cells. Colocalization of SFV proteins and LC3 was measured using the Manders’ coefficients tool in the coloc2 plugin of ImageJ. Background fluorescence was subtracted, and regions of interest were drawn around cells to discount intercellular spaces. These regions of interest were used to mask for correlation testing.

### Real-time quantitative PCR

RNA was extracted from cells using RNAzol® RT (Sigma-Aldrich, UK) according to the manufacturer’s instructions and suspended in DEPC-treated water. A total of 500 ng RNA was transcribed to cDNA using the iScript^TM^ cDNA Synthesis kit (Bio-Rad, UK) according to the manufacturer’s instructions and run under the following conditions: 25 °C for 5 min, 42 °C for 30 min and 85 °C for 5 min. Quantitative (q)PCR was performed using the iTaq^TM^ Universal SYBR® Green Supermix (Bio-Rad, UK). A total of 20–25 ng of cDNA template was used per reaction, and samples were run on a CFX96 Touch^TM^ system (Bio-Rad, UK). All qPCR primers are detailed in Table S1 (available in the online Supplementary Material) and were designed using Primer3. Gene expression was calculated by the 2^∆∆^Ct method after normalization to two housekeeping genes, *β*-actin and GAPDH.

### Data analysis, visualization and statistics

Data was tabulated and assessed using Prism 7.0 (GraphPad, USA). Figures were made using Prism software, and statistical testing via ANOVA with Tukey HSD post hoc testing and two-tailed T-test were also performed using Prism software. Results are dominantly expressed as mean±sem as detailed in figure legends. *P*-values ≤0.05 were noted for significance and are demonstrated by incrementing asterisks. Data acquired from fluorescent microscopy images were analysed using ImageJ (NIH, USA).

## Results

### Autophagy is upregulated only at the late stages of SFV infection

**Fig. 1.**
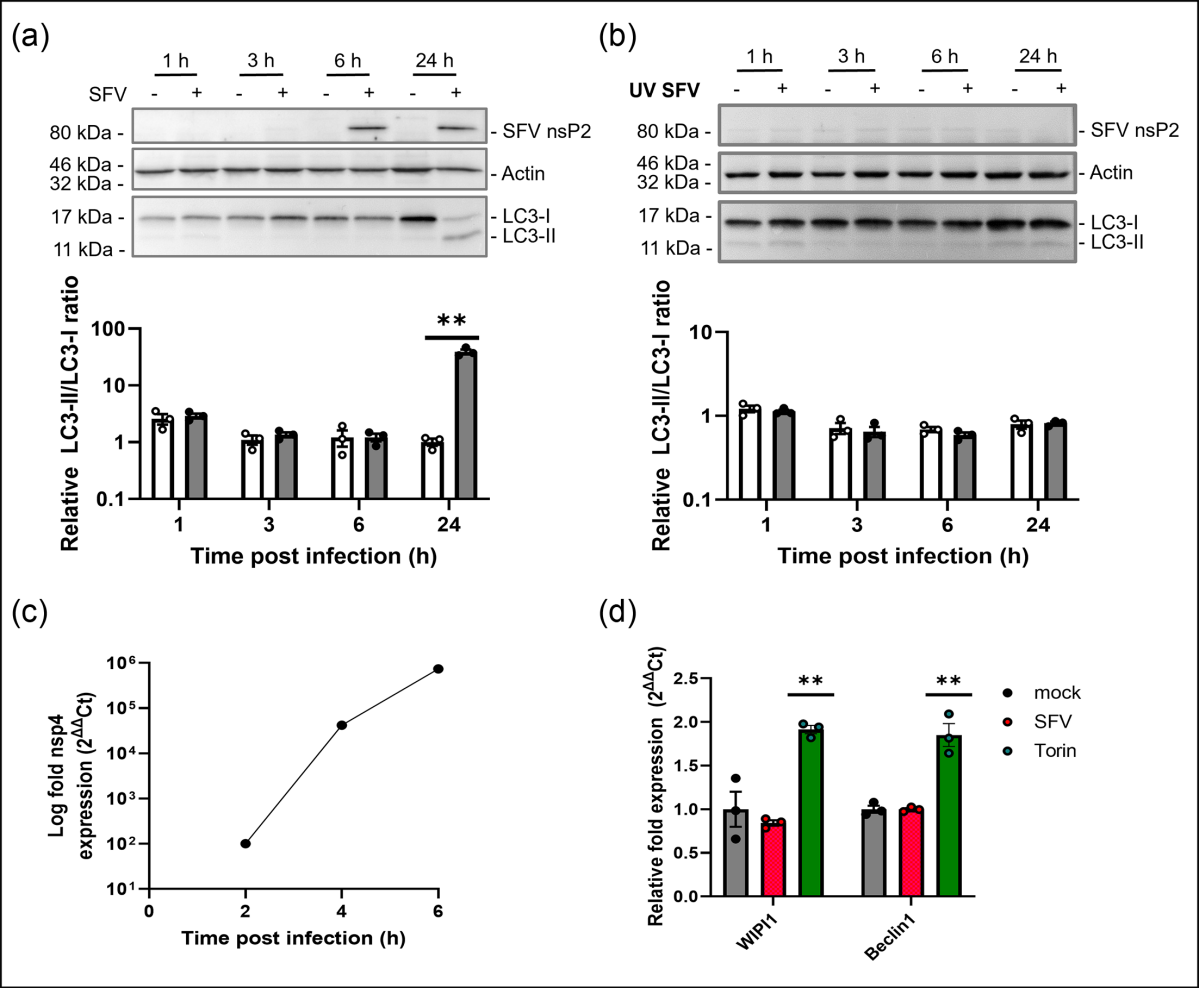
Analysis of autophagy regulation during infection of neurons. SK-N-SH cells were infected with (a) SFV (MOI=1), (b) UV-inactivated SFV or mock-infected and lysates were collected at specified time points. The LC3-II to LC3-I ratio was quantified using densitometric data of immunoblots probed for LC3, actin and SFV nsP2. Graphs represent densitometric data of LC3-II to LC3-I levels expressed as mean±se. (c) SK-N-SH cells were infected with SFV (MOI=1), and RNA was extracted at 2, 4 and 6 hpi. The relative fold expression of SFV nsP4 was examined using real-time qPCR. Expression was calculated using the 2^∆∆^Ct method and normalized to the housekeeping genes, *β*-actin and GAPDH. (d) Cells were infected with SFV (MOI=10) for 3 h or treated with 2 µM torin for 4 h. Relative fold expression of autophagy-related genes, WIPI1 and Beclin-1, was analysed by real-time qPCR as above. Graphs are shown as mean±se. Statistical significance is denoted as ** (*P*<0.001) (*n*=3).

Autophagy was measured during SFV infection of human neuronal cells by following the translocation of the microtubule-light chain associated protein LC3-I from the cytosol to the autophagosome membrane. This results in the production of LC3-II which can be detected by Western blot and generation of LC3 puncta in the cytosol. Western blot analysis (Fig. 1a) showed that there was no increase in LC3-II to LC3-I ratio in virus-infected cells when compared with mock-infected controls up to 6 h post-infection (hpi), but there was a profound increase in LC3-II at 24 hpi (39.11±3.73-fold increase, *n*=3) (Fig. 1a). This change in LC3-II was not observed when cells were infected with UV-inactivated SFV (Fig. 1b). Hence, the upregulation of autophagy at the later stages of infection was dependent on SFV replication, and this was confirmed in another human neuronal cell line, IMR-32 cells (Fig. S1).

The time of onset of LC3-II conversion was compared with the kinetics of viral genome replication by following the expression of RNA encoding nsp4 by real-time qPCR (Fig. 1c). Levels of nsp4 grew exponentially, several hours before increased levels of LC3-II. This was also confirmed in IMR-32 cells (Fig. S1C).

The activation of autophagy was also assessed by analysis of WIPI1 and Beclin-1 mRNA expression [30]. WIPI1 and Beclin-1 expression increased after induction of autophagy by torin, but little change in expression was detected in SK-N-SH or IMR-32 cells following virus infection for 3 h at a high MOI [10] (Figs 1d and S1D).

### Early infection with SFV suppresses basal autophagic flux

**Fig. 2.**
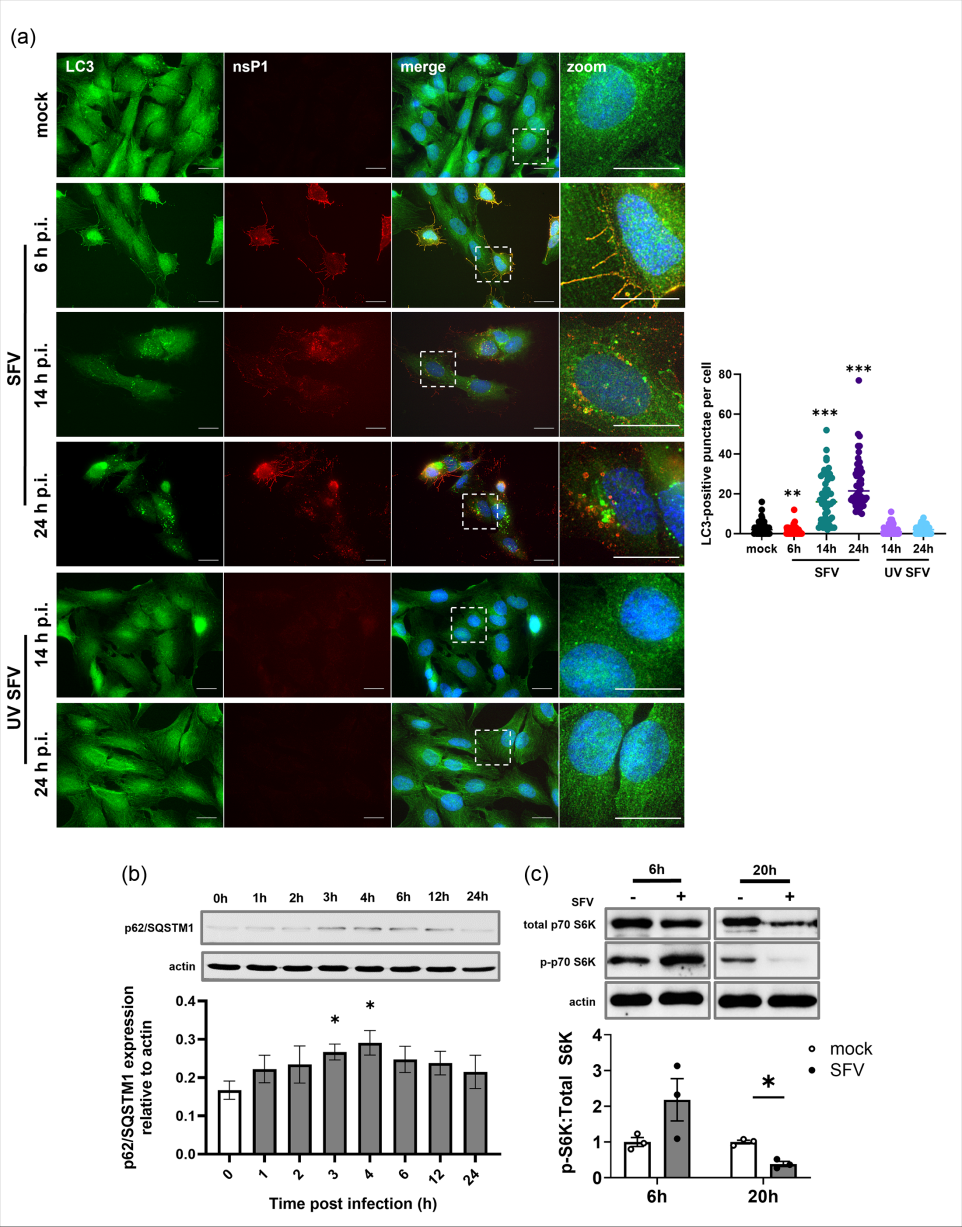
Quantification of autophagic activity during SFV infection of neurons. (**a**) LC3-positive punctae were quantified in mock, SFV and UV SFV-infected SK-N-SH cells at indicated time points. Cells were labelled with antibodies against LC3 (green), SFV nsP1 (red) and counterstained with DAPI (blue). The graph shows the number of LC3-positive punctae per cell±se (*n*=51). (**b**) SK-N-SH cells were infected with SFV (MOI=1), and lysates were collected hourly. Expression of p62/SQSTM1 and actin loading control was analysed by Western blot. (c) SK-N-SH cells were infected with SFV (MOI=1) for 6 and 20 h. Whole-cell lysates were analysed by Western blot with antibodies against total p70S6K, phosphorylated p70S6K and actin loading control. Images show representative blots. Graphs show densitometric analysis of blots (*n*=3) expressed as mean+se. Statistical significance is denoted as *P<0.05; **P<0.001; ***P<0.0001.

We assessed the production of LC3 puncta following SFV infection (Fig. 2a). After 6 h of infection with SFV, the number of autophagosomes per cell was significantly reduced from the basal level of autophagosomes present in mock-infected cells (1.31±0.31 vs. 3.07±0.51, respectively). At the later time points of 14 and 24 h, however, there was a six- and eightfold increase in the number of autophagosomes present, respectively. This increase was not seen in cells infected with UV-inactivated SFV (Fig. 2a). This suggests that SFV replication suppresses basal autophagy in neuronal cells at early times and upregulates autophagy between 6 and 14 hpi.

The delivery of material to autophagosomes can be measured by following the degradation of autophagy cargo binding proteins such as p62/SQSTM1. Fig. 2b shows that levels of p62/SQSTM1 increased during the first 4 h of infection; this again suggested that autophagy was suppressed early during infection. Levels of p62/SQSTM1 reduced back to near-control levels at 12 and 24 hpi, indicating that this accumulation was only short-lived during the early stage of infection. Modulation of autophagy can also be followed by analysing the activity of mTOR, which acts as a nutrient-sensing kinase to inhibit autophagy and promote protein synthesis. The p70 ribosomal protein S6 kinase (p70S6K) is downstream of mTOR, and increased mTOR activity leads to higher levels of phosphorylated p70S6K. Fig. 2c shows that SFV infection of neuronal SK-N-SH cells increased phosphorylation of p70S6K 2-fold at 6 hpi, whereas at 20 hpi, phosphorylated p70S6K decreased. These results suggest a dynamic regulation of mTOR activity during SFV infection that correlates with effects on autophagy.

### SFV infection inhibits the production of autophagosomes by torin or CHQ

**Fig. 3.**
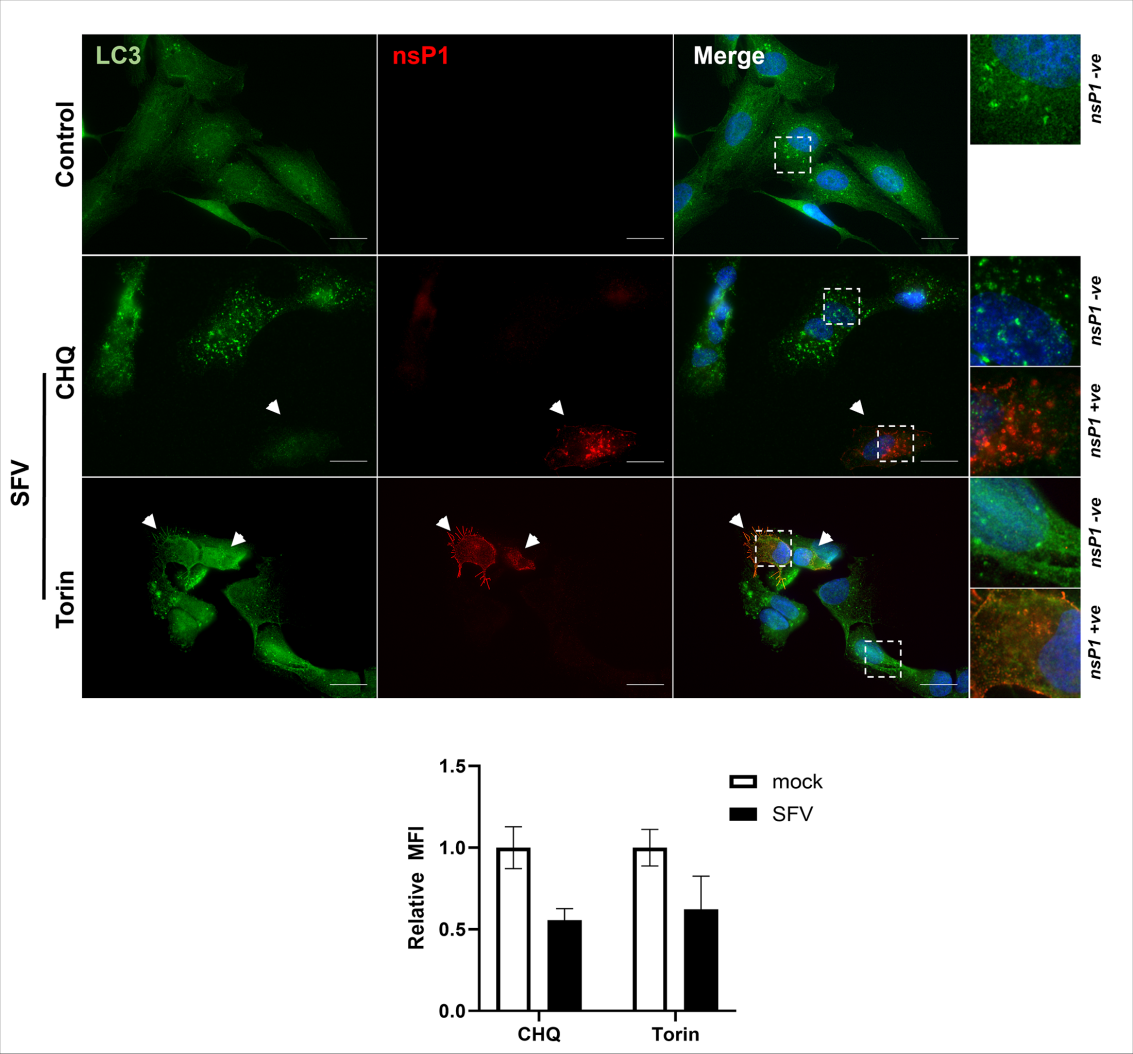
The effect of autophagy inducers on neurons after 4 h of infection. SK-N-SH cells were infected with SFV (MOI=0.1) for 4 h and then treated with 100 µM CHQ or 2 µM torin for 2 h. Cells were then fixed and probed with antibodies for LC3 (green), SFV nsP1 (red) and counterstained with DAPI (blue). Scale bars=20 µm. Hatched boxes represent zoomed regions of nsP1 positive or nsP1 negative cells. White arrows indicate nsP1 positive cells. The graph shows MFI relative to the uninfected cells in the same sample±se.

Cells infected with SFV were incubated with torin to activate autophagy through inhibition of mTOR. Fig. S2A shows that incubation of cells with Torin for 2 h activated autophagy as indicated by increased numbers of LC3 puncta (Fig. S2A) However, when torin was added to cells infected with a low MOI of SFV, autophagosomes were only generated in cells negative for nsP1, and autophagosomes were absent from infected cells expressing nsP1 (Fig. 3, arrows). CHQ inhibits the maturation of autophagosomes and completion of the autophagy pathway by raising lysosomal pH and inhibiting autophagosome–lysosome fusion. This causes an accumulation of autophagosomes which can be viewed by immunofluorescence staining of LC3. Incubation with CHQ for 2 h results in the accumulation of autophagosomes (Fig. S2A). In infected samples, however, CHQ increased numbers of LC3 puncta only in cells negative for nsP1 staining (Fig. 3), but these were absent in infected cells (Fig. 3, arrows). Indeed, even infected cells near uninfected cells showed no accumulation of autophagosomes, while uninfected cells showed a marked increase in autophagosome accumulation. Analysis of MFI showed that LC3 intensity was higher in uninfected cells after incubation with both CHQ and torin. These data further demonstrate an active suppression of autophagy during the first 6 h of SFV infection.

### SFV replication complexes colocalize with LC3-positive punctae at later stages of infection

**Fig. 4.**
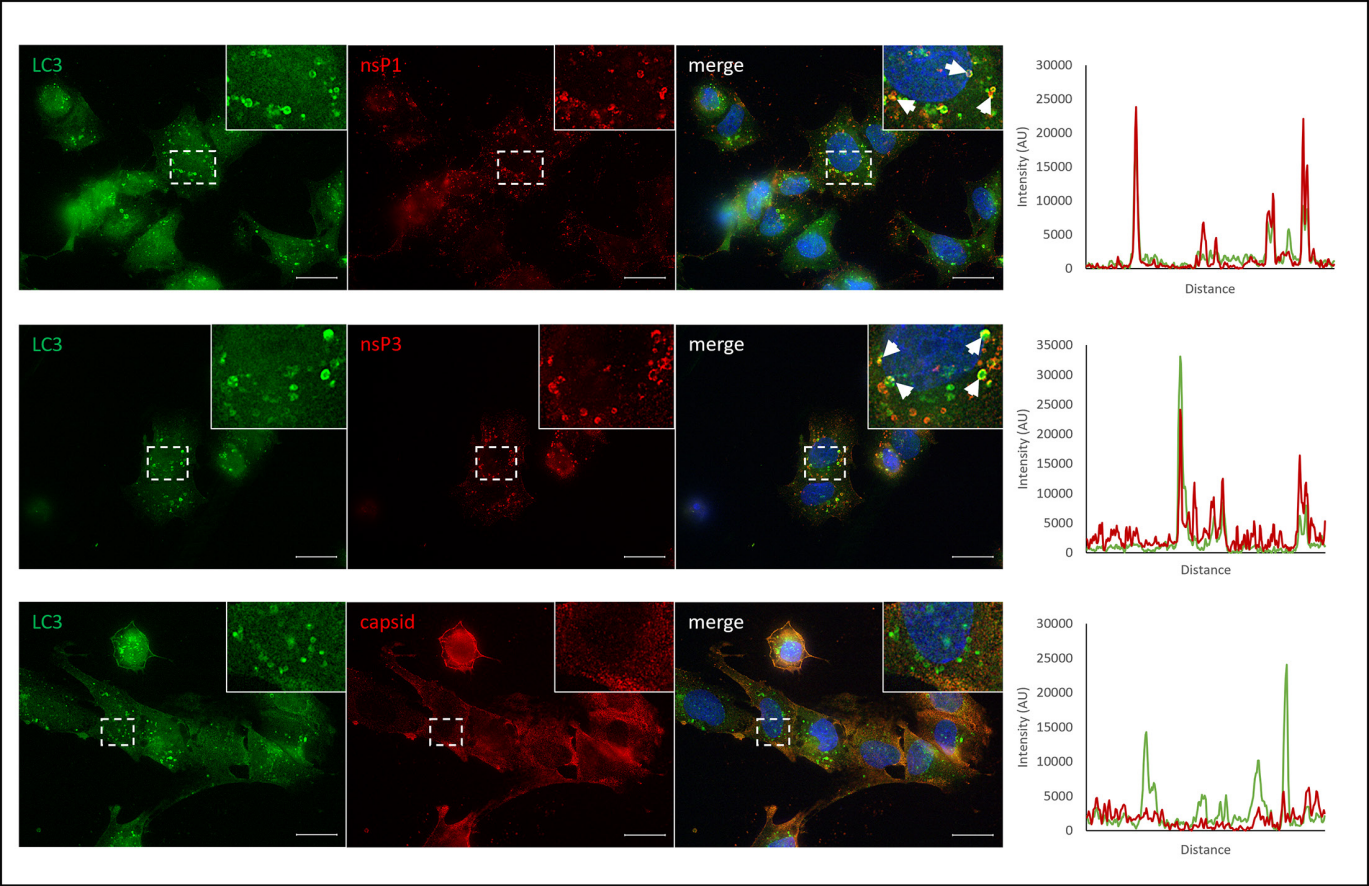
Distribution of viral proteins and autophagosomes. SK-N-SH cells were infected with SFV (MOI=1) for 24 h. Cells were then fixed and labelled with antibodies against LC3 (green); nsP1, nsP3 or capsid (red); and counterstained with DAPI (blue). White-hatched boxes represent regions of higher magnification (inset). Colocalization of LC3 punctae and SFV proteins is shown by white arrows. Scale bars=20 µm. Graphs show representative intensity profiles taken from cell cross sections.

It has previously been shown that LC3 punctae accumulate during the later stages of SFV infection of human osteosarcoma (HOS) cells due to an inhibition of autophagosome fusion with lysosomes [17]. This accumulation could suggest that SFV utilizes autophagosome membranes for sites of virus replication when viral production reaches a peak in the host cell. We, therefore, hypothesized that SFV replication complexes would colocalize with LC3-positive membranes at the late stages of infection. Cells were infected with SFV for 24 h and labelled with antibodies against LC3 and virus nonstructural proteins nsP1 and nsP3 which together form the virus replication complexes. Both nsP1 and nsP3 showed a strong overlap with LC3-positive punctae. Correlation analysis indicated positive levels of colocalization for both nsP1 (correlation coefficient 0.74) and nsP3 (correlation coefficient 0.63). It was notable that not all nsP1 and nsP3 staining was localized with LC3 punctae. However, the majority of LC3 punctae were positive for nsP1 or nsP3 (Fig. 4). This provided evidence that autophagosome membranes may be used by SFV for sites of replication complexes.

It was also assessed whether autophagosome membranes played a role in the packaging and formation of mature virions. To examine this, cells were infected with SFV for 24 h and labelled with antibodies against LC3 and the SFV capsid protein. There was no localization between LC3 and the SFV capsid protein (Fig. 4), and correlation analysis showed no colocatization between SFV capsid and LC3 (correlation coefficient 0.09). This provides further evidence to support the use of autophagosome membranes in the formation of replication complexes and not in virus assembly and maturation.

### Autophagy upregulation reduces viral yields and enhances cell survival

**Fig. 5.**
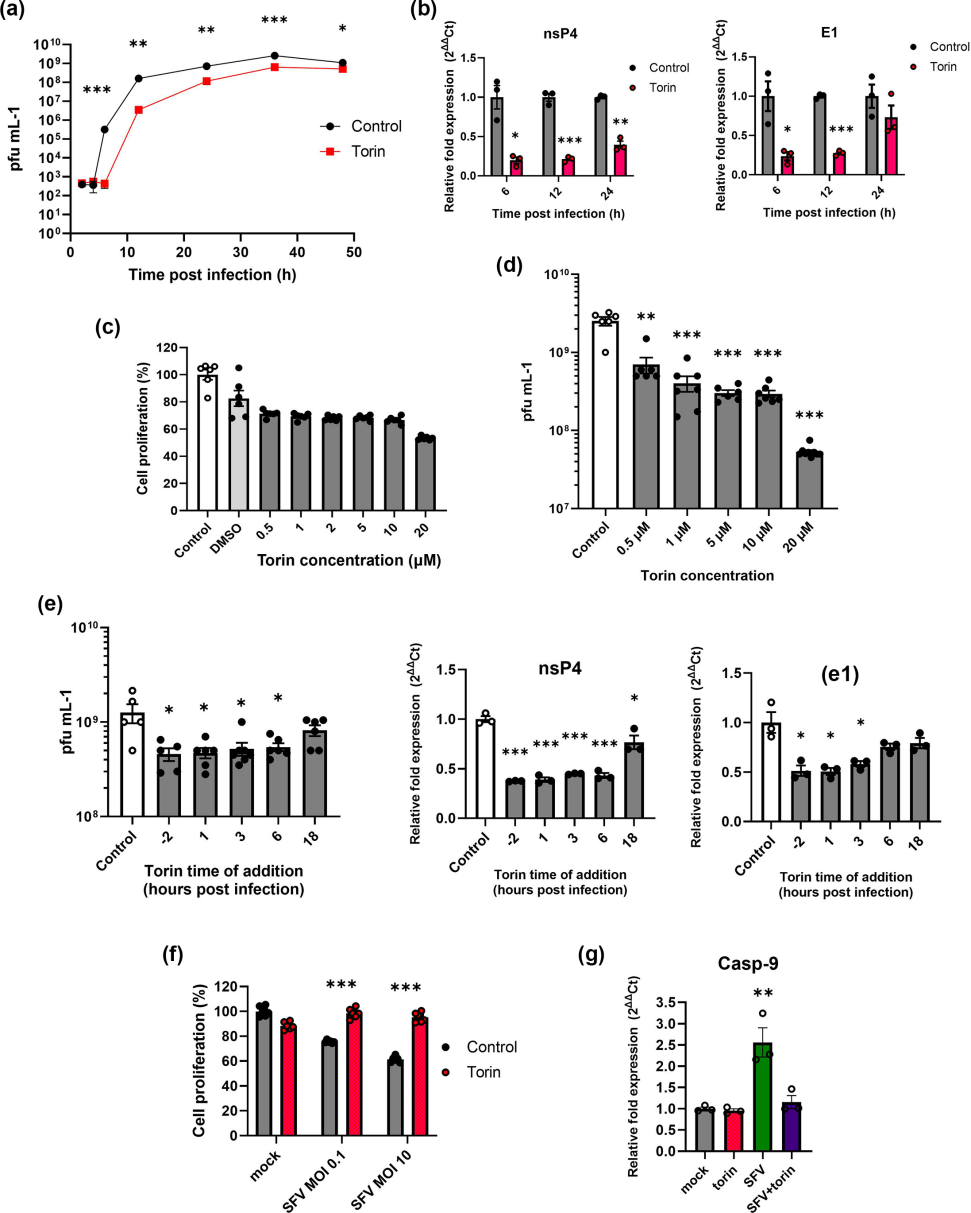
Analysis of torin induction of autophagy during SFV infection. SK-N-SH cells were infected with SFV (MOI=0.1) after pre-treatment 4 h prior to infection with 2 µM torin or control nutrient media. Virus titre was determined by (a) plaque assay or (b) real-time qPCR at specified time points. (c) SK-N-SH cells were incubated in torin at the indicated concentrations or DMSO alone for 24 h. Cell proliferation was measured by MTT assay. (d) SK-N-SH cells were infected with SFV (MOI=0.1) in the presence or absence of torin at the indicated concentrations. Infectious virus was titred at 24 hpi by plaque assay (*n*=6). (e) SK-N-SH cells were infected with SFV (MOI=0.1). 2 µM torin was added at indicated time points. Infectious virus was measured by plaque assay at 24 hpi (*n*=6). Gene expression of SFV nsP4 and E1 was measured by real-time qPCR (*n*=3). (f) SK-N-SH cells were infected with SFV (MOI=0.1) or 10 for 22 h in the presence or absence of 2 µM torin. Cell proliferation was measured by MTT assay. The graph shows means % survival±se relative to mock-infected cells (*n*=6). (g) SK-N-SH cells were incubated in control media or with 2 µM torin for 2 h. Cells were then infected with SFV (MOI=0.1) for 24 h. Caspase-9 expression was measured by real-time qPCR. Gene expression levels were normalized to *β*-actin and GAPDH (*n*=3). Values are expressed as mean±se relative to untreated control cells. Asterisks denote statistical significance (*P<0.05, **P<0.001, ***P<0.0001).

The suppression of autophagy during the early stages of infection suggests that SFV limits the formation of autophagosomes to establish infection more efficiently. We, therefore, hypothesize that the chemical induction of autophagy prior to infection would lower virus yields during the early stages of infection. We first confirmed that pre-treatment and continuous exposure to torin allowed the formation of autophagosomes to continue through the first 6 h of SFV infection. Levels of infectious virus released were profoundly reduced in cells that were incubated with torin when compared with control cells. This was observed at all times up to 48 hpi; however, the reduction became less significant at later time points (Fig. 5a). Concurrently, SFV genome levels were also reduced in cells incubated with torin, an effect that was also less pronounced at the later time point of 24 hpi (Fig. 5b).

Cell proliferation in response to torin was measured by MTT assay. Cells incubated with a range of torin concentrations showed a 20–30% reduction in proliferation after 24 h although this was only statistically significant at the highest concentration of 20 µM with a 40% reduction in comparison to cells incubated in complete media (Fig. 5c). As torin stocks were suspended in DMSO, we compared these to a DMSO-only control and found the reduced proliferation in response to torin was much less, suggesting that the reduction in proliferation was largely due to the presence of DMSO. This reduction in cell proliferation was also much lower than the reduction seen in viral titres, suggesting that reduced cell proliferation was not the main cause of the antiviral effect observed. Torin incubation also reduced virus yields in a concentration-dependent manner. Cells were infected in the presence of a range of torin concentrations, and cell supernatants were analysed at 24 hpi by plaque assay (Fig. 5d). Correlative analysis showed a significant negative correlation that as torin concentration increased, infectious virus production decreased (*r*=−0.815). These results were also confirmed in the alternate neuroblastoma cell line IMR-32 (Fig. S2).

The differential regulation of autophagy we show here suggests that chemically inducing autophagy with torin would have a more prominent effect when administered early in infection when compared with later time points of infection. To test this hypothesis, torin was added to SFV-infected cells at different time points, and virus yields were examined at 24 hpi. Plaque assay of cell supernatant showed that when torin was administered during the first 6 h of infection, virus production was significantly lower after 24 hpi than control untreated cells. However, when torin was administered at a later time point (18 hpi), there was no significant reduction in the virus released at 24 hpi (Fig. 5e). Similarly, expression of SFV nsP4 and E1 genes was also more significantly reduced when administered earlier during infection (at −2 h, 0.38±0.00 and 0.051±0.04 (*P*≤0.001 and *P*≤0.05), respectively). Correlative analysis showed that there was a significant positive correlation between the time of torin addition and the expression of both nsP4 (*r*=0.9) and E1 (*r*=0.875). These data provide more evidence that autophagy is suppressed during the early stages of infection, as the antiviral effect of torin is highest during this time.

Next, we examined the effect of increased autophagy on cell survival during infection. Although incubation with torin reduced virus yields, this was not due to a lower level of cell survival during infection. Cells were infected in the presence or absence of torin for 22 h at low or high MOIs, and then cell proliferation was measured by MTT assay. Although uninfected cells incubated with torin showed a minor decrease in proliferation, the opposite effect was observed in infected cells. After 24 hpi, cells incubated with torin showed a higher level of cell proliferation than cells infected in the absence of torin (98.50±1.64, 95.20±1.60 vs. 75.60±0.44, 61.35±0.97, for MOI 0.1 and 1, respectively) (Fig. 5f). Interestingly, a higher MOI caused a further decrease in cell proliferation in untreated cells, but there was still no decrease in cells incubated with torin. While it is known that cell death during SFV infection is by apoptosis, we hypothesize that increasing autophagy with torin reduces the level of apoptosis to prolong cell survival. The expression of the apoptosis marker caspase-9 was, therefore, examined by real-time qPCR during infection in the presence or absence of torin. Torin incubation had no effect on caspase-9 expression in the absence of infection. Infected cells incubated with torin expressed lower levels of caspase-9 after 24 hpi when compared with infected cells in the absence of torin (0.63±0.02, *P*≤0.05) (Fig. 5g), an effect that was also observed in IMR-32 cells (Fig. S2E). The intrinsic effect of autophagy on cell survival during infection was also shown in MEFs deficient for the major autophagy gene ATG5. In these cells, autophagosomes cannot form even when treated with torin (Fig. S3A). During infection, cell proliferation was profoundly decreased when compared with wild-type MEFs (Fig. S3B). Taken together, these data show that autophagy provides a protective effect during SFV infection and suggests that increasing autophagy with torin prolongs cell survival by reducing apoptosis during infection.

## Discussion

Here, we show several indications of dynamic regulation of autophagy between the early and late stages of SFV infection of neurons. Infection of neuronal cells with SFV and UV-inactivated SFV did not result in any increase in LC3-I to LC3-II conversion up to 6 h of infection. Similarly, herpes simplex virus, Kaposi’s sarcoma-associated herpesvirus and African swine fever virus (ASFV) show no change in % protein degradation or % cells undergoing autophagy. This is later reported to be due to the active suppression of autophagy by virally encoded genetic products [31,33]. This LC3-II expression was, however, increased at later stages of infection with SFV but not with UV-inactivated SFV, showing that increased autophagosome formation at later stages of infection is dependent upon virus replication. This is concurrent with the study by Eng *et al*. [17] in which they show that the accumulation of autophagosomes at later stages of infection in non-neuronal cells is dependent upon the expression of SFV glycoproteins.

Gene expression analysis of autophagy-related genes including WIPI1 and Beclin-1 is a useful measure of autophagosome formation in neurons as well as non-neuronal cells [30]. Levels of WIPI1 and Beclin-1 expression were increased by the autophagy inducer torin but were no different in SFV-infected cells early in infection. This further demonstrates no autophagy upregulation in early SFV infection. Further to this, we show an accumulation of p62/SQSTM1, an accepted marker of autophagic flux, during the first 4 h of infection. This protein binds to ubiquitinated cytosolic components which are then targeted to autophagosomal membranes. The targeted p62/SQSTM1 then binds with LC3-II and is degraded upon maturation of the autophagosome and subsequent fusion with the lysosome [34]. Inversely, the accumulation of p62/SQSTM1 can indicate a disruption in the conventional lysosome fusion and degradation functions of autophagy [14,35]. The accumulation shown here suggests that part of the autophagic pathway is suppressed and complete maturation and degradation of autophagosomes is inhibited.

The increase of p62/SQSTM1 in the absence of any increase in LC3-II or expression of autophagy-related genes suggests no increase in autophagosome formation in early infection. To support this evidence, the number of autophagosomes per cell was quantified via immunofluorescence microscopy. In neurons that were infected with SFV, there was a noticeable reduction in the number of autophagosomes after 6 h of infection when compared to uninfected controls. Whereas, later in infection, autophagosome numbers increased, showing that while autophagy is suppressed during the first 6 h of infection, there is then a switch to increased autophagosome formation. As there are a variety of physiological effects of autophagy, a basal level of autophagosome formation always occurs [36]. In virus-infected neurons, however, this basal level of formation was suppressed. The mechanism of this autophagy suppression was hypothesized to be due to increased mTOR activity. Analysis of the levels of phosphorylated p70S6K provides a reliable assessment of mTOR activity. Early in infection, mTOR activity is greater as shown by increased levels of p70S6K phosphorylation, whereas, after 20 hpi, levels of phosphorylated p70S6K are dramatically reduced. There was a notable reduction in the levels of total p70S6K which can be attributed to the inhibition of host protein synthesis by SFV during infection. However, the ratio of phosphorylated p70S6K is a reliable indication of the levels of mTOR activity. This is concurrent with evidence from Thaa *et al.* [18] that showed SFV infection for up to 8 h increased phosphorylation of p70S6K even in the presence of mTOR inhibitors such as rapamycin or torin. This further supports evidence for a switch between autophagy suppression early during infection to autophagy upregulation at later stages. Notably, some RNA viruses such as hepatitis C virus and SINV have been shown to increase not only autophagosome formation but indeed complete autophagic flux [14,37]. However, several viruses have evolved mechanisms to suppress the formation or maturation of autophagosomes and have been reported to negatively regulate autophagy through the targeting and binding of Beclin-1 [32,38, 39]. Other viruses have developed strategies to inhibit autophagosome maturation and block degradation [40,43].

We also report a further extent to the suppression of autophagy during early infection with SFV. When we examine a few hours into SFV infection of neurons, we show that treatment with the autophagosome-inducing chemical torin and the autophagosome-accumulating chemical CHQ have no effect on cells that were positive for SFV nsP1. Notably, bystander cells in the same sample that did not show any viral protein presence were subject to autophagosome accumulation by both torin and CHQ treatments in a manner indistinguishable from those of the control cells. This shows that the suppressive effect of SFV infection on autophagy during the first 6 h is enough that incubation with the autophagy inducers tested here is ineffective. Resistance to autophagy-inducing drugs has previously been reported in non-neuronal cells infected with human cytomegalovirus [35] in much the same manner as ASFV [44]. These results suggest an active suppression of autophagy by SFV at the very early stages of infection.

The upregulation of autophagy shown at later stages of infection suggests that autophagosomes may be used to enhance virus replication. We show that autophagosome membranes localize with both nsP1 and nsP3, which are indicative of the virus replication complexes but do not localize with the structural capsid protein. Due to this, we hypothesize that autophagy is suppressed to allow more efficient replication of the viral genome. There is currently limited evidence of interactions between SFV protein and autophagy-related proteins through immunoprecipitation experiments, but there is evidence of direct interaction between the capsid proteins of CHIKV and SINV and p62/SQSTM1 [9,14] although the replication of these viruses is enhanced by increased autophagy. Future work should explore direct interactions of SFV proteins with autophagy-related proteins and the specifics of virus replication in association with autophagosome membranes by using dsRNA-specific antibodies or through the purification of active replication complexes as reported by [6]. It is at later stages of infection that there is a switch from genome replication to protein production. The later stages of infection in which autophagosomes are accumulated may be a response to an increasing need for membranes as sites for replication complexes as the viral yield within the cell increases. As viral protein production requires intracellular membranes or replication complexes that accumulate around cytopathic vacuoles at the MTOC, it is possible that the accumulation of autophagosomes at later stages of infection that have previously been reported [17] is a response to this switch in the virus life cycle. There is also a possibility that autophagy may affect virus assembly and egress, although this is an aim for future experimental studies.

We further characterized the switch in autophagy regulation by SFV through viral yield experiments. Induction of autophagy prior to infection with torin was profoundly unfavourable to SFV infection. Release of infectious virus was lower in cells incubated with torin; however, this effect was less significant at time points later than 24 hpi. Over the course of the infection, intracellular SFV genome yield was markedly reduced in cells that were pre-treated with torin. Time-of-addition experiments also showed that torin incubation was more detrimental to virus production and intracellular virus genome levels when administered earlier in infection. It is reasonable to assume from this data that in order to establish a prominent infection of SFV in neuronal cells, autophagy must be suppressed to promote the early stages of genome replication. Autophagy induction was also shown to enhance cell survival later in infection and reduced caspase-9 expression, which indicates lower levels of apoptosis. However, it is unclear from the data presented here whether this reduction is a direct result of autophagy induction or a result of reduced SFV production. The protection of autophagy during virus infection has also been shown during SINV infection *in vitro* and *in vivo* [14] although a differential regulation of autophagy by SINV during early and late stages of infection has not been examined. It is also worth considering the impact of innate immune factor expression that may be altered by incubation with autophagy inhibitors. It is widely reported that alphaviruses induce the expression of innate immune genes [45]. The expression of these genes should also be examined during infection alongside incubation with autophagy inducers such as torin. However, care should be taken when interpreting these results as changes in innate immune gene expression may come as a result of reduced virus infection and not as a direct result of autophagy induction itself.

Taken together, the results reported here provide an insight into the close relationship between the early autophagy pathway and the different stages of SFV infection in neuronal cells. It has been previously reported that SFV infection of non-neuronal cells results in the accumulation of autophagosomes at later stages of infection [17]. In the present study, however, we examine human neuronal cell lines at both early and later stages of SFV infection. In summary, we have identified a difference in the regulation of autophagy in the early period of SFV infection compared to the later stages in neuronal cells. We report that the treatment of neuronal cells with chemical inducers of autophagy limits SFV replication and infectious virus production and enhances cell survival. This provides a more detailed understanding of the initial stages of virus infection in neurons as well as increasing the understanding of the whole process of infection of neurons with SFV. The effect of autophagy-inducing drugs on viral yield also highlights the importance of autophagy as a therapeutic treatment for RNA virus infection and the importance of early treatment.

## supplementary material

10.1099/jgv.0.002086 Uncited Supplementary Material 1.

## Abbreviations

ASFV: African swine fever virus
CHIKV: Chikungunya virus
CHQ: chloroquine
CPE: cytopathic effect
HIFC: heat-inactivated foetal calf serum
HOS: human osteosarcoma
hpi: hours post-infection
MEM: minimum essential medium
MFI: mean fluorescence intensity
MOI: multiplicity of infection
MTOC: microtubule-organizing centre
mTOR: mammalian target of rapamycin
MTT: 3-[4,5-Dimethylthiazol-2-yl]-2,5-diphenyltetrazolium bromide
NEAA: non-essential amino acids
nsP1: nonstructural protein 1
qPCR: quantitative PCR
SFV: Semliki Forest virus
SINV: Sindbis virus
