# Supplementary material for: Dynamic regulation of autophagy during Semliki Forest virus infection of neuroblastoma cells
Source: J Gen Virol. 2025 Mar 5;106(3):002086. doi: 10.1099/jgv.0.002086 (PMC11882037; doi:10.1099/jgv.0.002086)
Supplement: Uncited Supplementary Material 1. [file jgv-106-02086-s001.pdf]

Dynamic regulation of autophagy during Semliki Forest virus infection of neuroblastoma cells

Robert J Stott-Marshall[1&2]*, Craig McBeth[3] and Thomas Wileman[4]

8. Supplementary Figures and tables

Table S1. Primers used in RT-qPCR analysis

	**Sequence (5′ → 3′)**		
**Target**	**Forward**	**Reverse**	**Reference**
GAPDH	TTCACCACCATGGAGAAGGC	GGCATGGACTGTGGTCATGA	(46)
β-actin	AGAGGGAAATCGTGCGTGAC	CAATAGTGATGACCTGGCCGT	(46)
SFV E1	CGCATCACCTTCTTTTGTG	CCAGACCACCCGAGATTTT	(47)
SFV nsP4	CCGCCCCGTGTACTCCCCTA	AGCTTCGCCGGGCAGAATGT	
WIPI1	GCACATCCCTAGCAACTGGAA	CGTTCATCTGCCGTGGTTTT	
Beclin 1	TAGACCAGCTGGACACTC	CTTGCGGTTCTTTTCCAC	
Casp-9	TGCTGAGCAGCGAGCTGT T	AGCCTGCCCGCTGGAT	

Company Limited by Guarantee l Registered in England No. 1039582 l Registered Office Charles Darwin House, 12 Roger Street, WC1N 2JU, UK
Registered as a Charity: 264017 (England & Wales); SC039250 (Scotland)

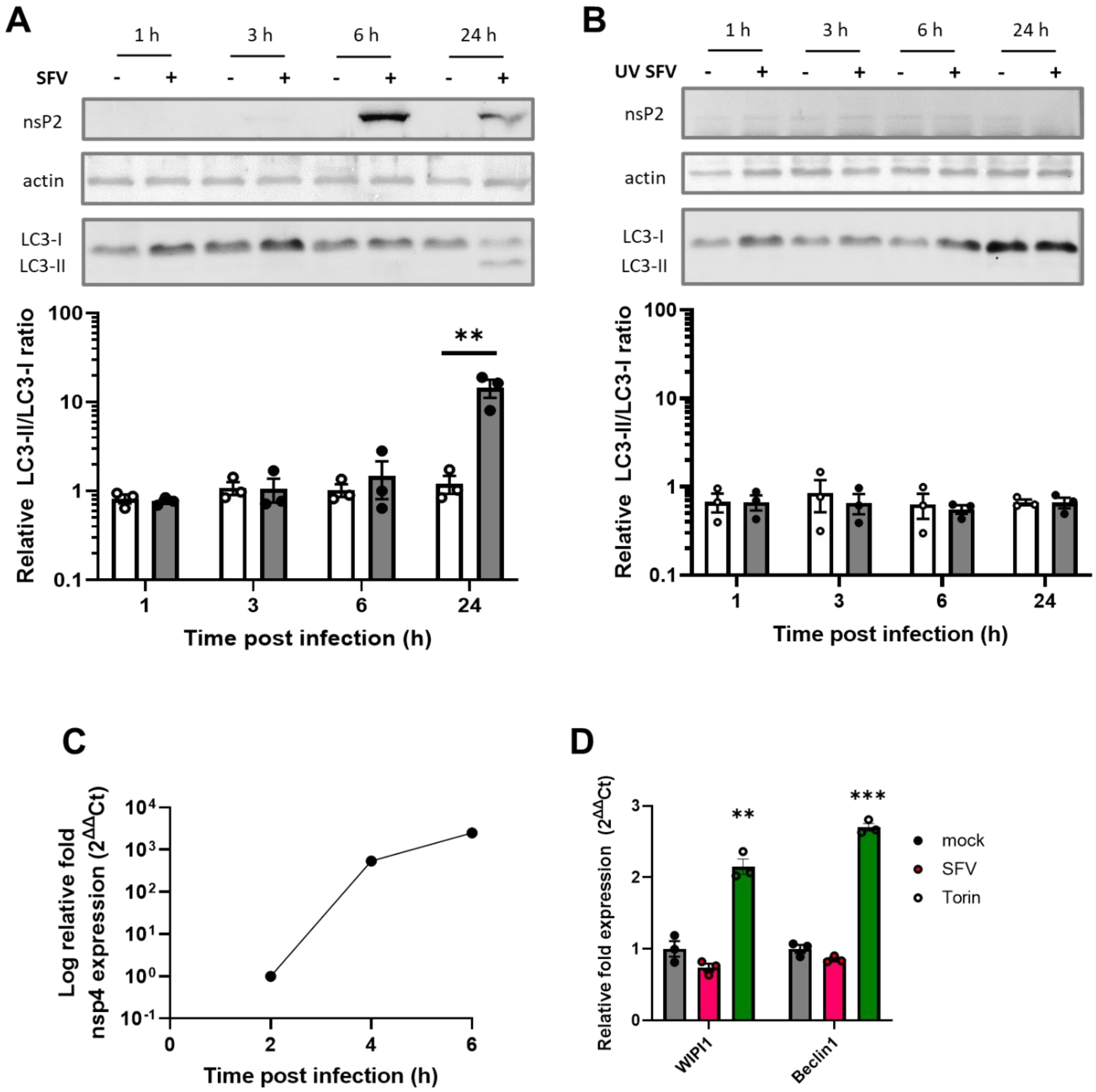

Figure S1. IMR-32 cells were infected with **(A)** SFV (MOI=1) or **(B)** UV inactivated SFV. Whole cell lysates were analyzed by western blot at specified time points with antibodies against SFV nsp2, LC3 and actin as a loading control. Graphs show mean ratio of LC3-II expression to LC3-I normalized to an uninfected control. **(C)** IMR-32 cells were infected as above and expression of SFV nsP4 mRNA was analyzed by RT-qPCR relative to β-actin and GAPDH housekeeping genes. **(D)** IMR-32 cells were infected as above for 3h or incubated with 2µM torin for 4h. Expression of genes WIPI1 and Beclin1 were analyzed relative to housekeeping genes β-actin and GAPDH. Graphs are expressed at means ±SE (n=3). Asterisks denote statistical significance ($p<0.05$ = *, $p<0.001$ = **, $p<0.0001$ = ***)

7

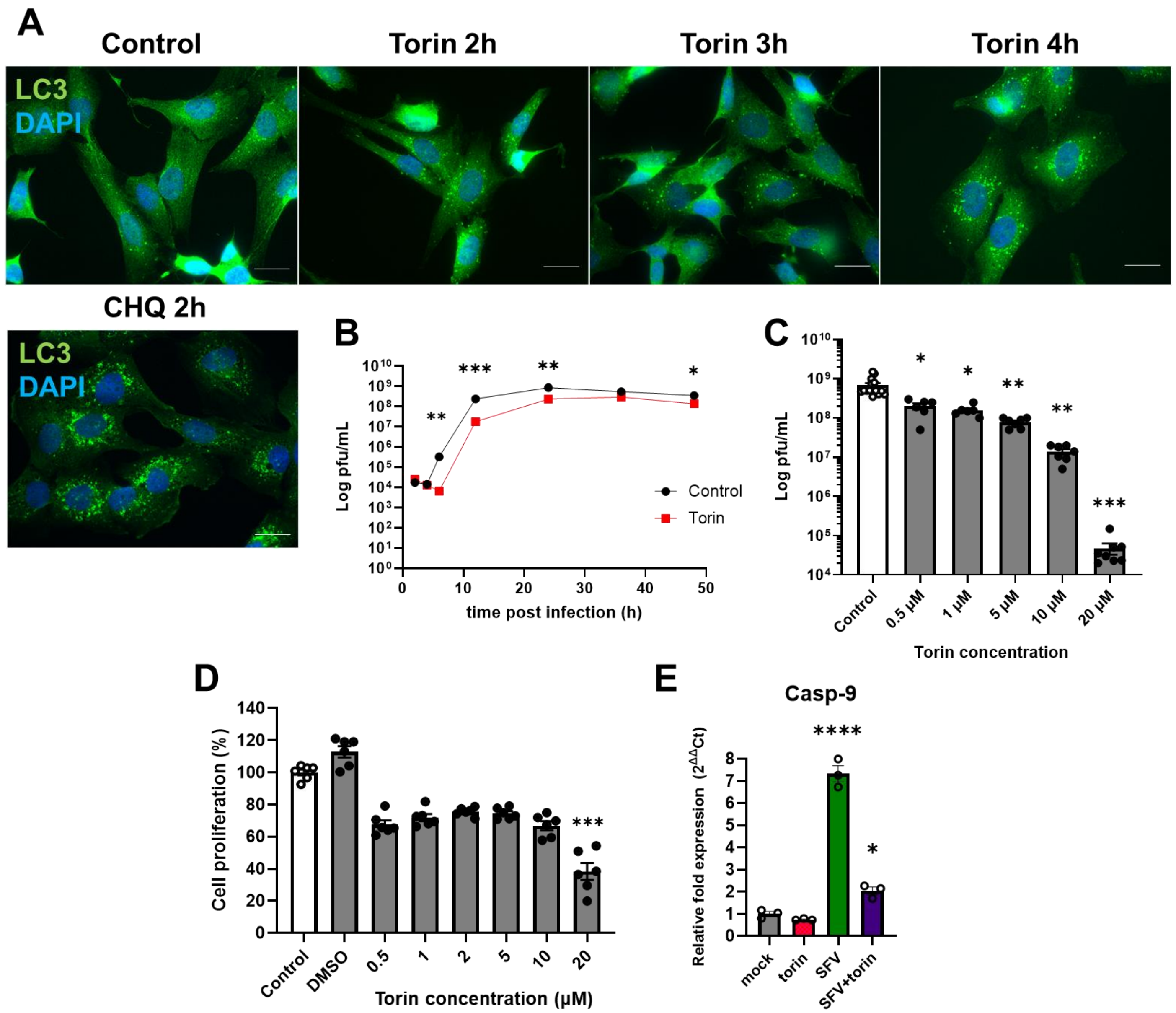

Figure S2. (A) SK-N-SH cells were incubated on coverslips with control media, 2µM torin up to 4h or 100µM CHQ for 2h then processed with an antibody against LC3 (green) to assess autophagosome formation. **(B)** IMR-32 cells were infected with SFV (MOI=10) after pre-treatment 4h prior to infection with 2µM torin or control nutrient media and supernatant was collected at time points indicated. Virus titre was determined by plaque assay. **(C)** IMR-32 cells were infected with SFV (MOI=0.1) in the presence or absence of torin at the indicated concentrations. Infectious virus was titered at 24hpi by plaque assay (n=6). **(D)** IMR-32 cells were incubated in torin at the indicated concentrations or DMSO alone for 24h. Cell proliferation was measure by MTT assay. **(E)** IMR-32 cells were incubated in control media or with 2µM torin for 2h. Cells were then infected with SFV (MOI=0.1) for 24h. Caspase 9 expression was measured by RT-qPCR. Gene expression levels were normalized to β-actin and GAPDH (n=3). Asterisks denote statistical significance (p<0.05 = *, p<0.001 = **, p<0.0001 = ***)

8

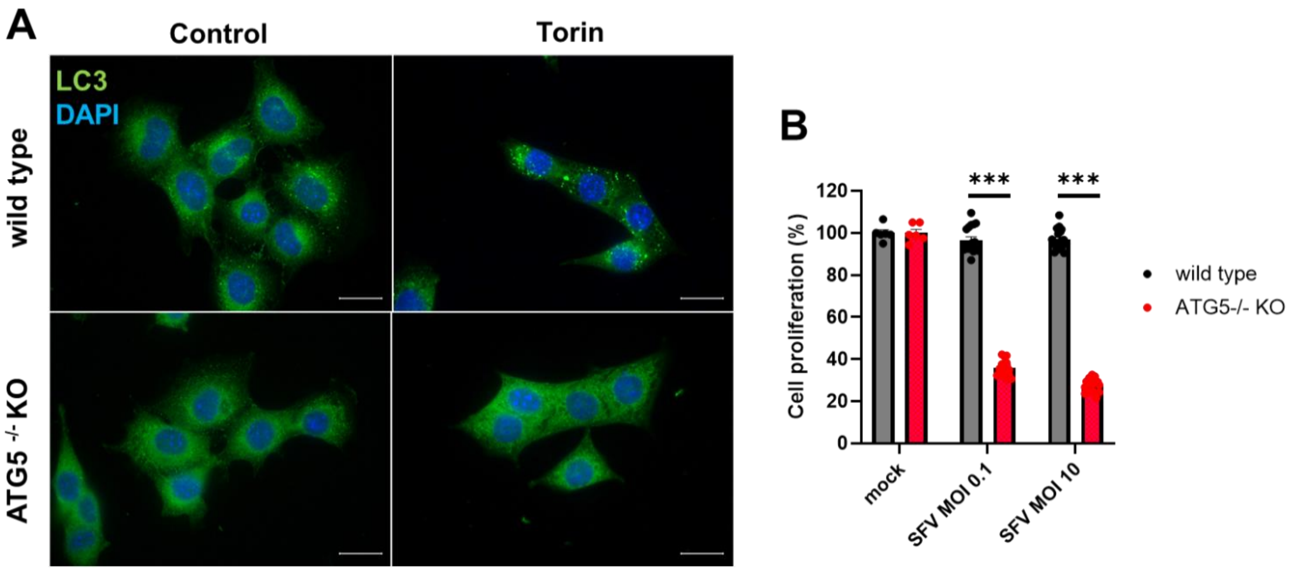

Figure S3. (A) Wild-type or Atg5 knockout MEFs were incubated on coverslips with 2µM torin for 4h. samples were then processed with an antibody against LC3 (green) to assess autophagosome formation. **(B)** Wild-type or Atg5 knockout MEFs were infected with SFV (MOI=0.1 or 10) for 22h. Cell proliferation was then measured by MTT and is expressed as a percentage relative to the mock infected controls (n=6). Values are expressed as mean ±SE relative to untreated control cells. Asterisks denote statistical significance (p<0.0001 = ***)

9
